# Editorial: Rising stars in child mental health and interventions

**DOI:** 10.3389/frcha.2024.1500765

**Published:** 2024-10-25

**Authors:** Yael Dvir, Ujjwal Ramtekkar

**Affiliations:** ^1^ Psychiatry/Child and Adolescent Psychiatry, UMass Chan Medical School, Worcester, MA, United States; ^2^ School of Medicine, University of Missouri, Columbia, MO, United States

**Keywords:** child mental health, interventions, school, psychotherapy, emotional regulation, caregivers

**Editorial on the Research Topic**
Rising stars in child mental health and interventions

We are pleased to introduce Frontiers Research Topic—*Rising stars in child mental health and interventions*. This collection of articles includes 12 original papers authored by 81 authors and an intentionally broad array of topics highly relevant to the field of child and adolescent psychiatry. It is no secret that children and adolescents’ mental health and wellbeing have received significant public attention in recent years, following documented increases in depression, anxiety, and neurodevelopmental disorders. As articulated in the US Surgeon General Advisory on protecting youth mental health, the COVID-19 pandemic enhanced youth's pre-existing challenges and increasing rates of psychological distress, especially in already vulnerable populations. The advisory concluded that maintaining health children and families requires all of society to take part in this effort (1). The United Nations unicef and the World Health Organization have also highlighted youth mental health as a global challenge, which affects young people in every part of the world, causing significant suffering (2, 3). As such, we are encouraged that the themes of this research topic suggest that new and exciting opportunities to improve children and adolescents’ mental health are occurring in many setting where youth are cared for, and that enhancing the scientific knowledge in mental health and allied fields is *a priori*ty. We would like to reflect on some of the themes that emerged for us while editing this topic.

Children and adolescent spend many hours in school and educational environments, and educators and other professional staff at school are important in noticing and reporting on any concerns in both typically developing youth and those with diagnosed developmental or behavioral health conditions. Using educational settings to assess social skills and predict depression has been an important component of the work of Krygsman et al. who report on *depression symptoms, communication and cooperation skills, and friendship: longitudinal associations in young Norwegian children*. Their findings suggest a relationship between depressive symptoms in preschool, the development of early social skills and friendships, and later childhood depression. In a study delivering an intervention in an elementary school setting, Leger-Goodes et al. describe *feasibility, acceptability, and perceived benefits of a creative arts intervention for elementary school children living with speech, language and communication disorders*. Their pilot work suggests acceptability of this model by educational teams as well as potential benefits for student's emotional expression.

While the evidence base for psychotherapeutic approaches to youth's behavioral health challenges are expanding, one challenge for practicing clinicians is that most of these interventions are studied in academic settings. Therefore, the work done by the two following groups of researchers adapting and testing treatment protocols to and in diverse settings, internationally or in correctional settings, are highly important in bridging these gaps. Dozio et al. reported on *comparing the effectiveness of narrative therapy and EMDR-GTEP protocols in the treatment of post-traumatic stress in children exposed to humanitarian crises*. Their study which was conducted with children in the Central African Republic suggests that both protocols are effective in reducing symptoms of traumatic exposure and can be conducted by trained paraprofessionals. Given that many parts of the world are currently experiencing humanitarian crises situations for various reasons, this is a highly relevant study. Similarly, while dialectical behavioral therapy has a significant evidence base for the treatment of emotional dysregulation in adolescents with several mental health diagnosis, it has not been shown to be effective or feasible in correctional settings. Folk et al. have shown in their pilot study that *comprehensive dialectical behavior therapy for adolescents in a juvenile correctional treatment center* is a promising intervention that can be implemented with success.

Smith et al. are offering an important contribution to the evidence-based approaches to serious mental illness in youth, reporting on *alternative psychopharmacologic treatments for pediatric catatonia: a retrospective analysis*.

Several studies included in this collection provide insights into how tailored interventions can reduce anxiety, stress, emotional dysregulation, and other mental health issues among youth and their caregivers.

Caregivers play a central role in managing the mental health of anxious children, and recent studies emphasize the need to support caregivers’ well-being alongside that of their children. Raftery-Helmer et al. highlight the effectiveness of Acceptance and Commitment Therapy for Parents of Anxious Children (ACT-PAC), which reduces caregivers’ stress and cognitive challenges linked to child anxiety. By addressing these caregiver-specific issues, the study shows improvements in both caregiver mental health and child anxiety, underscoring the interconnectedness of their well-being.

Similarly, Huffman et al. found that when children undergo digital mental health interventions (DMHIs) such as behavioral coaching and therapy, caregivers also experience significant benefits. Caregivers reported improvements in sleep and reduced stress levels, demonstrating that addressing children's mental health has positive effects for the entire family, emphasizing the value of family-centered mental health care that includes caregivers in the therapeutic process.

As climate change becomes an increasing concern, studies are beginning to focus on the mental health impacts of ecological issues, particularly among young people. Malboeuf-Hurtubise et al. explore the growing phenomenon of eco-anxiety in youth, showing that positive psychology interventions can help mitigate feelings of hopelessness by fostering resilience, hope, and meaning-making. By encouraging young people to transform their anxiety into proactive engagement with environmental issues, this approach offers an innovative way to support youth mental health in the face of global challenges.

Maternal health, particularly during pregnancy and early childhood, has a profound impact on the long-term mental health of children. Jalnapurkar et al. emphasize that maternal physical and mental health conditions, as well psychosocial challenges, are linked to higher rates of anxiety and depression in extremely low gestational age newborns after 15 years. Their findings highlight the importance of early interventions aimed at improving maternal health, which in turn can significantly reduce the risk of psychiatric disorders in their children. This research underscores the need for preventive mental health strategies that extend beyond the child to include maternal and early-life factors.

When it comes to managing emotional dysregulation and ADHD in children, multidimensional interventions are showing promising results. Villagomez et al. investigate the role of broad-spectrum micronutrients (BSMs) in improving emotional regulation in psychiatric conditions like ADHD and mood disorders. Their research suggests that BSMs offer a complementary approach to traditional psychiatric treatments, contributing to more effective emotional management. In another study, Luo et al. explored the combination of behavior modification training and EEG biofeedback to treat children with ADHD. Their research demonstrated significant improvements in core symptoms such as hyperactivity, impulsivity, and inattention, particularly when both methods were used together. This comprehensive approach, integrating behavioral and neurological interventions, highlights the potential of multi-modal treatments for managing complex psychiatric conditions like ADHD.

The findings from these studies highlight the need for holistic, multidimensional approaches to mental health care while considering the well-being of both children and caregivers.
